# Steering on Degrees of Freedom of 2D Van der Waals Heterostructures

**DOI:** 10.1002/smsc.202100033

**Published:** 2021-10-13

**Authors:** Hui-Zhen Zhang, Wen-Jing Wu, Lin Zhou, Zhen Wu, Jia Zhu

**Affiliations:** ^1^ National Laboratory of Solid State Microstructures College of Engineering and Applied Sciences School of Physics Key Laboratory of Intelligent Optical Sensing and Manipulation Ministry of Education Jiangsu Key Laboratory of Artificial Functional Materials Nanjing University Nanjing 210093 P. R. China; ^2^ Department of Electrical Engineering The Pennsylvania State University University Park Pennsylvania 16802 USA

**Keywords:** degrees of freedom, 2D materials, 2D van der Waals heterostructures

## Abstract

2D heterostructures have garnered tremendous attention for potential applications in electronics and optoelectronics. Heterostructures can be constructed by assembling individual atomically thin layers of 2D materials into integrated devices, which involves three primary degrees of freedom (DOFs), i.e., Lego‐like basic building blocks, out‐of‐plane stacking order, and in‐plane twist‐angle alignment. By steering the DOFs of 2D materials, devices and structures such as artificial Shockley junction, quantum wells, and superlattices can be conveniently established based on well‐developed fabrication and/or assembly techniques, beneficial for next‐generation ultracompact information technologies. Herein, the recent progress on constructing the artificial atomic structures by taking advantage of three primary DOFs is overviewed. An outlook of the challenges and future developments is presented as well. Future advancements in the rational construction of complex devices and artificial heterostructures are also suggested.

## Graphical abstract

2D atomic crystal materials are most attractive candidates for next‐generation optoelectronics because of their unique mechanical, electronic, and optical properties. Extraordinarily, the heterogeneous assembly of 2D material structures has evidenced plenty of intriguing physical properties. From the viewpoint of the precise fabrication, this review summarizes the most important degrees of freedom and their potential figures of merit. Finally, the review provides a future perspective on the artificial heterostructures of 2D atomic crystals.

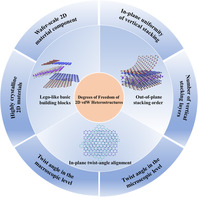

## 1 Introduction

**Scheme 1:**
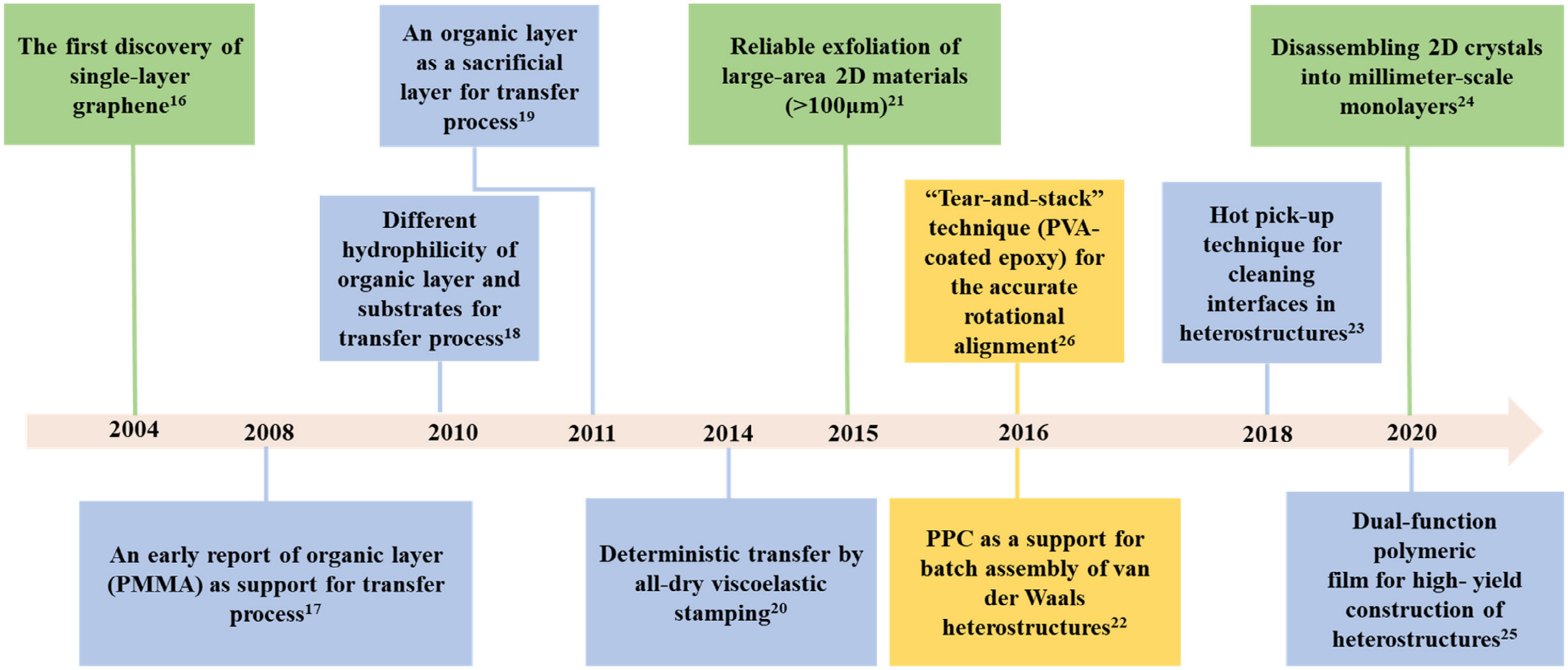
Timeline of important events in vdW heterostructures.

**Figure 1:**
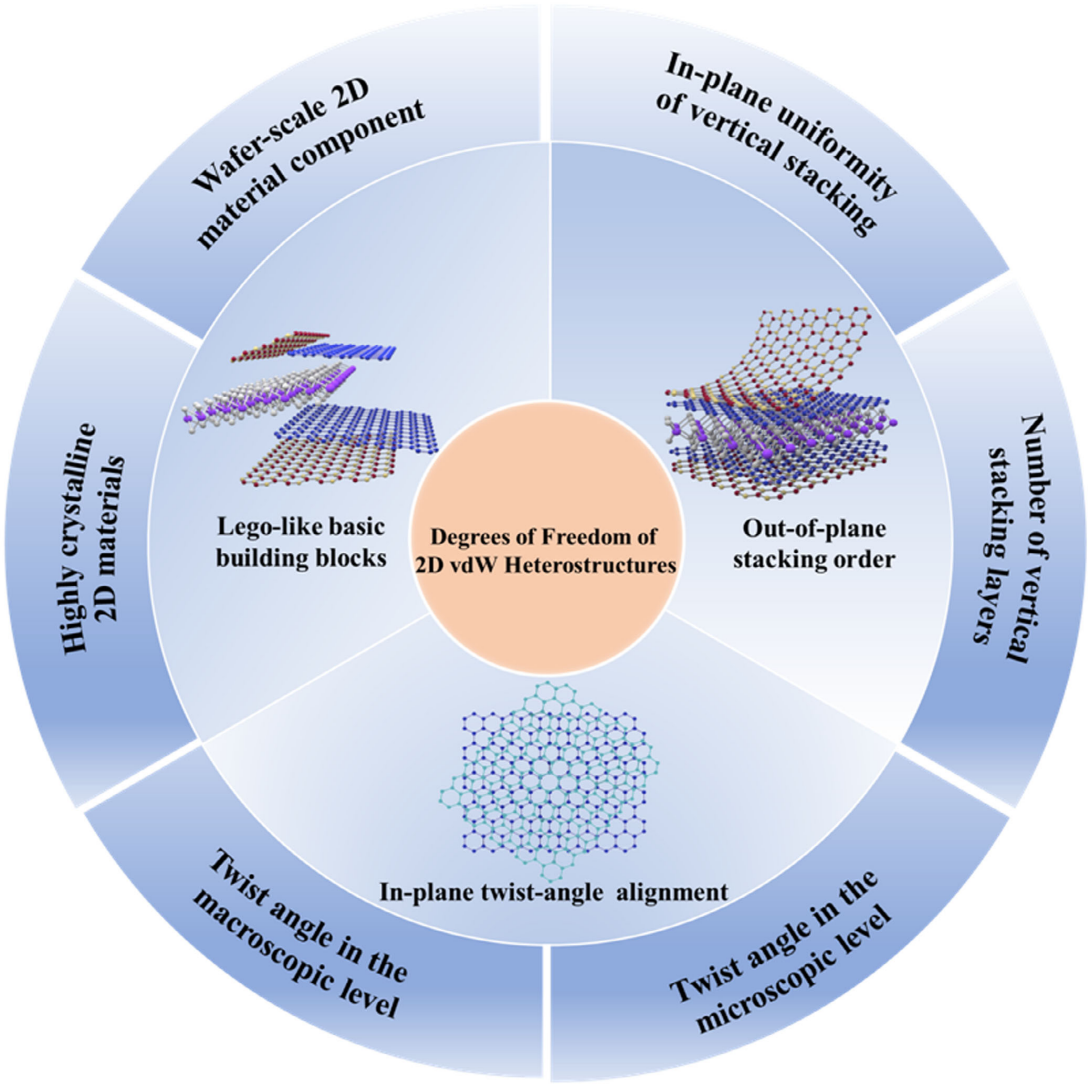
Heterostructures assembled with manipulating the DOFs of 2D materials. The three primary DOFs are “Lego‐like basic building blocks”, “out‐of‐plane stacking order,” and “in‐plane twist‐angle alignment.”

2D materials have recently received explosive attention because of their unique mechanical, electronic, and optical properties for flexible electronics and optoelectronics beyond traditional III–V semiconductor technologies.^[^ 1, 2, 3, 4, 5, 6 ^]^ With self‐assembly of 2D materials layer by layer like Legos, van der Waals (vdW) heterostructures of intriguing physical properties such as superfluidity and superconductivity can be constructed, inspiring tremendous interest in macroscopic quantum phenomena.^[^ 7, 8, 9, 10, 11, 12 ^]^ Unlike heterostructures made of conventional semiconductors, the electronic behaviors of vdW heterostructures can be engineered by the intrinsic building blocks and the interlayer alignment, which leads to the emerging field of twistronics. Taking transition metal dichalcogenides (TMDs), for example, when the TMD bulk crystals are thinned down to atomic layers, the 2D atomic crystals experience a phase transition from indirect to direct semiconductors.^[^ ^13^ ^]^ Such a phase transition can give rise to pronounced photoluminescence in optical frequency range determined by the bandgap and exciton binding energy. As they are stacked vertically as heterostructures, efficient electron–hole separation across the interface can be enabled via an ultrafast charge transfer process, leading to extremely long‐lived interlayer exciton with lifetime up to micron second time scale.^[^ ^14^ ^]^ Furthermore, by precisely controlling the in‐plane twist‐angle alignment between two TMD monolayers, both the intralayer and interlayer exciton emission can be tailored by the formation of moiré superlattice, which results in a lateral patterned potential landscape.^[^ ^15^ ^]^ In addition, 2D hexagonal boron nitride (h‐BN) can promote the carrier mobility of graphene‐based heterostructures up to 25 000 cm^2^ V^−1^ s^−1^.^[^ ^16^ ^]^ Inserting an atomic h‐BN layer into a MoS_2_–WSe_2_ heterostructure can tune interlayer coupling of charge carriers at the heterointerface and is expected to yield tunable optoelectronic properties.^[^ ^17^ ^]^ In addition, certain‐angle‐twisted graphene homobilayers have evidenced moiré‐based effects, which would remarkably change the electronic behavior of the heterostructures.^[^ ^10^ ^]^ Till now, a variety of methods have been reported in fabricating 2D vdW heterostructures. The key milestones in these developments are illustrated in Scheme 1.^[^ 18, 19, 20, 21, 22, 23, 24, 25, 26, 27, 28 ^]^ Generally speaking, the uniformity and integrity of the prepared heterostructures are highly dependent on the as‐used fabrication processes. Based on the evaluation of the step‐by‐step stacking flow path, one can disassemble the stacking processes into three crucial steps, selecting the target 2D material flakes (or called as Lego‐like basic building blocks), designing the out‐of‐plane stacking order of the heterostructures (for vertical microstructure definition), and the twist‐angle alignment between adjacent flakes (for lateral microstructure definition), which finally determines the electronic band structures and physical properties of the heterostructures. Based on the aforementioned logicality, the effectiveness of the manipulation of 2D heterostructures can be mainly ascribed to three DOFs, the Lego‐like basic building blocks, out‐of‐plane stacking order, and the in‐plane twist‐angle alignment. First, Lego‐like basic building blocks are defined as the 2D materials (like the isolated Lego blocks) used to stack target 2D heterostructures. Second, the out‐of‐plane stacking order refers to the vertical interfacial lattice of the stacked heterostructures, which can be further described as the number of 2D materials involved, interfacial arrangement, and the uniformity between layers of the vertical structure. The third DOF of 2D heterostructures is the in‐plane twist‐angle alignment, illustrated as the variability of heterostructures in the 2D material plane. The variability are the accuracy of twist angle in the macroscopic level and the uniformity of twist angle in the microscopic level between layers. All these advancements reveal that electronic and/or optoelectronic properties of 2D vdW heterostructures are highly dependent on three primary DOFs from the viewpoint of the microfabrication level, which refers to Lego‐like basic building blocks, out‐of‐plane stacking order, and in‐plane twist‐angle alignment, as shown in **Figure** **1**.

In the experiment, the flexibility originating from the Lego‐like basic building blocks of 2D materials also brings up great challenges in constructing ideal 2D heterostructures. A couple of merit figures in terms of requirements on the fabrication processes are required for these DOFs. 1) As 2D materials for stacking vdW heterostructures, Lego‐like basic building blocks of considerable crystal quality and achievable area are critical in terms of the functionality as well as application requirements. Point defects in Lego‐like basic building blocks can strongly affect the electric and optical properties of the constructed heterostructures.^[^ 29, 30, 31, 32, 33 ^]^ 2) Out‐of‐plane stacking order with clean atomic layers and close‐packed configuration plays a second critical role. Contaminants between layers can deteriorate or even break down the device's performance by decoupling the different atomic layers.^[^ ^34^ ^]^ 3) In‐plane twist‐angle alignment with high precision and spatial uniformity is crucial. Precise control of interlayer alignment between atomic layers in both macroscopic and microscopic levels should be carefully evaluated in the magic angle based on physical effects and/or devices.^[^ 10, 11 ^]^ Therefore, it is necessary to reevaluate the versatile fabrication technologies in terms of the proposed DOFs.

During past decades, by manipulating DOFs of 2D stacking materials, researchers reported vdW heterostructures with novel properties for fundamental exploration and future applications. A couple of reviews have classified the existing methods from the fabrication principles, potential applications, and so on.^[^ 35, 36, 37, 38, 39 ^]^ In this mini review, we would like to summarize the advancement in the fabrication processes of vdW heterostructures in terms of the DOFs and their figures of merit (FOMs) toward optoelectronic devices. First, we describe the primary DOFs and their critical requirements. Next, we review the state‐of‐art of different assembling methods which can manipulate the DOFs. Finally, we outlook the challenges and future developments in the construction of complex devices and artificial heterostructures.

## 2 Manipulating DOFs of vdW Heterostructures

### 2.1 Lego‐like Basic Building Blocks

**Figure 2:**
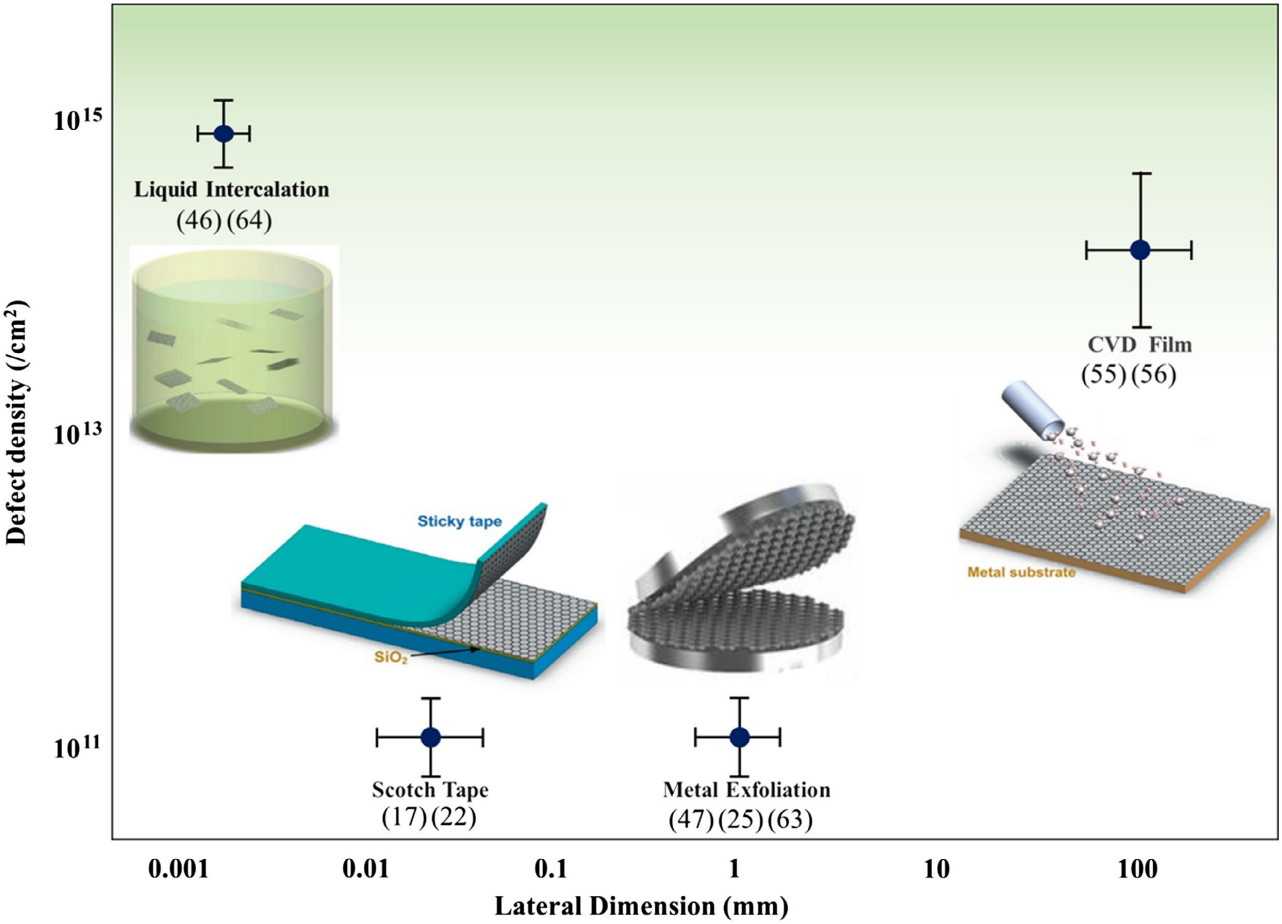
There are several methods of production of TMD monolayers, which allow a wide choice in terms of lateral dimension and defect density.^[^ 18, 23, 26, 47, 48, 56, 57, 63, 64, 143 ^]^ Metal Exfoliation: Reproduced with permission.^[^ ^62^ ^]^ Copyright 2020, American Chemical Society. Liquid Intercalation, Scotch Tape, CVD Film: Reproduced with permission.^[^ ^143^ ^]^ Copyright 2012, Elsevier Ltd.

As the basic building blocks for vdW heterostructures, 2D material flakes (monolayers most widely explored) are one of the most crucial components. Two of the most critical FOMs are the lateral size and defect density of the 2D material monolayers. In the past few years, large‐area 2D materials (monolayer or few layers in thickness) are extensively pursued to meet the requirements from point‐of‐use large scale devices such as transparent electrodes for display and electronic application integrated with silicon device flows.^[^ 40, 41, 42, 43, 44 ^]^ In the meantime, high‐quality 2D materials with low defect density, where defects origin from the exfoliation processes and/or fabrication processes of chemical vapor deposition (CVD), are highly desired due to the unique capability to explore the intrinsic physical effects under extreme conditions.^[^ 45, 46 ^]^ Defects of 2D materials for electronic devices should be effectively reduced as well. The past decades have evidenced the development of versatile fabrication methods toward large lateral area and/or low defect density 2D materials, both of which are highly dependent on the fabrication procedures.^[^ 47, 48 ^]^ There are four common fabrication methods, such as liquid intercalation, scope tape method, metal exfoliation method, and CVD method. A few‐layer 2D materials obtained by liquid intercalation are dispersed in a specific liquid.^[^ 49, 50 ^]^ However, 2D materials by this method are high‐density defect and several microns because the in‐plane covalent bonds can easily be broken due to mechanical force.^[^ 47, 51, 52 ^]^ CVD method has been widely used in the scale‐up production due to its highest control over preparation conditions, such as the temperature, pressure, and gas atmosphere of growth, which leads to higher purity and quality 2D materials compared with liquid intercalation method.^[^ 51, 53, 54 ^]^ However, the quality of 2D materials grown by CVD method is still inferior to that of 2D material nanoflakes exfoliated from bulk crystals due to the existence of defects, such as grain boundaries, wrinkles, impurities, and atomic vacancies.^[^ 55, 56, 57, 58 ^]^ The defect density of 2D materials exfoliated from bulk crystals is the lowest because of high quality.^[^ 55, 59, 60 ^]^ The advantages of large size and low defects make it the most promising approach to obtain a large area of monolayer materials by depositing metals to achieve stronger interaction with 2D materials.^[^ 61, 62 ^]^ Taking TMD monolayers, for example, the fabrication methods dependence of lateral dimension and defect density of 2D material monolayers is shown in **Figure** **2**. In the following section, we emphasize on most productive methods for obtaining large‐area and high‐quality 2D materials.

Since the first report of successful exfoliation of graphene in 2004 by Geim, several methods have been developed to produce monolayer 2D materials by top‐down approaches.^[^ 18, 26, 47, 48, 63 ^]^ The ion‐intercalation and liquid exfoliation can be used for scalable production of monolayers within a few micrometers size.^[^ 18, 49, 50, 52, 64 ^]^ Compared with the scotch tape‐based exfoliation method, metal‐assisted exfoliation method is more favorable for exfoliating monolayer 2D materials up to ~ centimeter size due to covalent‐like quasibonding between metals and 2D materials. The distinct breakthrough in large‐scale 2D building blocks has dramatically advanced the development toward point‐of‐use 2D devices. CVD is also an effective way to obtain wafer‐scale 2D materials.^[^ ^54^ ^]^ In the following section, we emphasize on most productive methods for obtaining large‐area and high‐quality 2D materials.

#### 2.1.1 Wafer‐Scale 2D Building Blocks

Large‐size monolayer samples are particularly important for practical application of 2D materials. Generally, metal‐assisted mechanical exfoliation and CVD method are most common strategies for large‐size 2D material preparation. The mechanical exfoliation is the most straightforward and universal method. In the mechanical exfoliation process, the vdW interactions play most critical roles between both bulk materials as well as between the outermost surface of 2D materials and substrates. It indicates a competition between the interlayer and interfacial forces and only when Γ_2D–Substrate_ > Γ_2D–2D_ can 2D flakes be separated from bulk materials.^[^ 55, 65 ^]^ Therefore, it is crucial to enhance the adhesion between the outermost surface of the 2D materials and substrates to obtain large‐area 2D flakes. In the traditional mechanical exfoliation, the unevenness of the bulk materials and the substrate easily leads to the bubbles at the interface, causing poor interfacial adhesion. The lateral size of samples prepared by this traditional method is limited within 10–50 μm^2^, and rarely exfoliated monolayer sample can be obtained successfully.^[^ 66, 67 ^]^ Huang et al. have improved this method for exfoliating large area monolayer samples with two crucial additional steps such as oxygen plasma cleaning and mild annealing treatment.^[^ ^23^ ^]^ Such treatments can enable strong adhesion of the outermost atomic layer to substrates, increasing the area of the exfoliated flakes by more than 50 times compared with the traditional exfoliation methods. Recently, some researchers used a metal‐assisted method to obtain centimeter‐scale monolayer 2D materials, which has greatly improved the development of the practical application.^[^ 26, 63, 68, 69, 70 ^]^ Covalent‐like quasibonding, formed between metals and 2D layers, balances a reasonably large Pauli repulsion and enhances adhesion between metals (such as Ni and Au) and 2D layers (Figure 2a). One of the important points is the flatness of the metal film of nanometer level, which makes the metal and the 2D material uniform adhere. Based on this method, Liu et al. disassembled 2D bulk materials into macroscopic monolayers with atomically flat gold tape with near‐unity yield (Figure 2b). And Huang et al. exfoliated monolayers, such as FeSe, PtTe_2_, and PdTe_2_, which are not accessible using other mechanical exfoliate methods. In addition, CVD method is a scalable and controllable way to grow large‐area 2D materials. CVD process includes nucleation and growth of 2D materials, which involves gaseous materials reacting on the surface of substrates and generating solid products deposited on the substrates. Continuous 2D films can be growing by mainly controlling the growth parameters such as temperature, chamber pressure, carrier gas flow rate, relative amounts of source materials, and source–substrate distance (Figure 2c). Senthilkumar et al. developed direct vapor phase growth method for large‐area MoS_2_ monolayers by carefully controlling the amounts of precursors of MoO_3_ and S powders in a catalyst‐free process.^[^ ^57^ ^]^ Li et al. and coworkers grew large‐area graphene films of the order of centimeters on copper substrates (Figure 2d).^[^ ^44^ ^]^ In previous work, graphene grown on Ni seemed to be limited by its small grain size, various layers at the grain boundaries due to the high carbon solubility of Ni.^[^ 40, 71 ^]^ It provided a pathway to grow self‐limited graphene films because of the low solubility of carbon in copper, which was beneficial for large‐area continuous single‐layer graphene. Zhou et al. reduced the graphene nucleation density by maintaining a catalytic inactive Cu_2_O layer during the initial nucleation stage.^[^ ^72^ ^]^ And the giant graphene single crystals ensure remarkable electronic properties with the expected quantum Hall effect and the highest carrier mobility up to 16 000 cm^2^ V^−1^ s^−1^. The mechanical exfoliation method can obtain 2D materials with nearly perfect lattice structure, limited by a small area previously. However, recently developed metal‐assisted process for obtaining wafer‐scale samples is a promising approach due to 2D materials of high quality at the same time.

#### 2.1.2 Highly Crystalline 2D Materials

**Figure 3:**
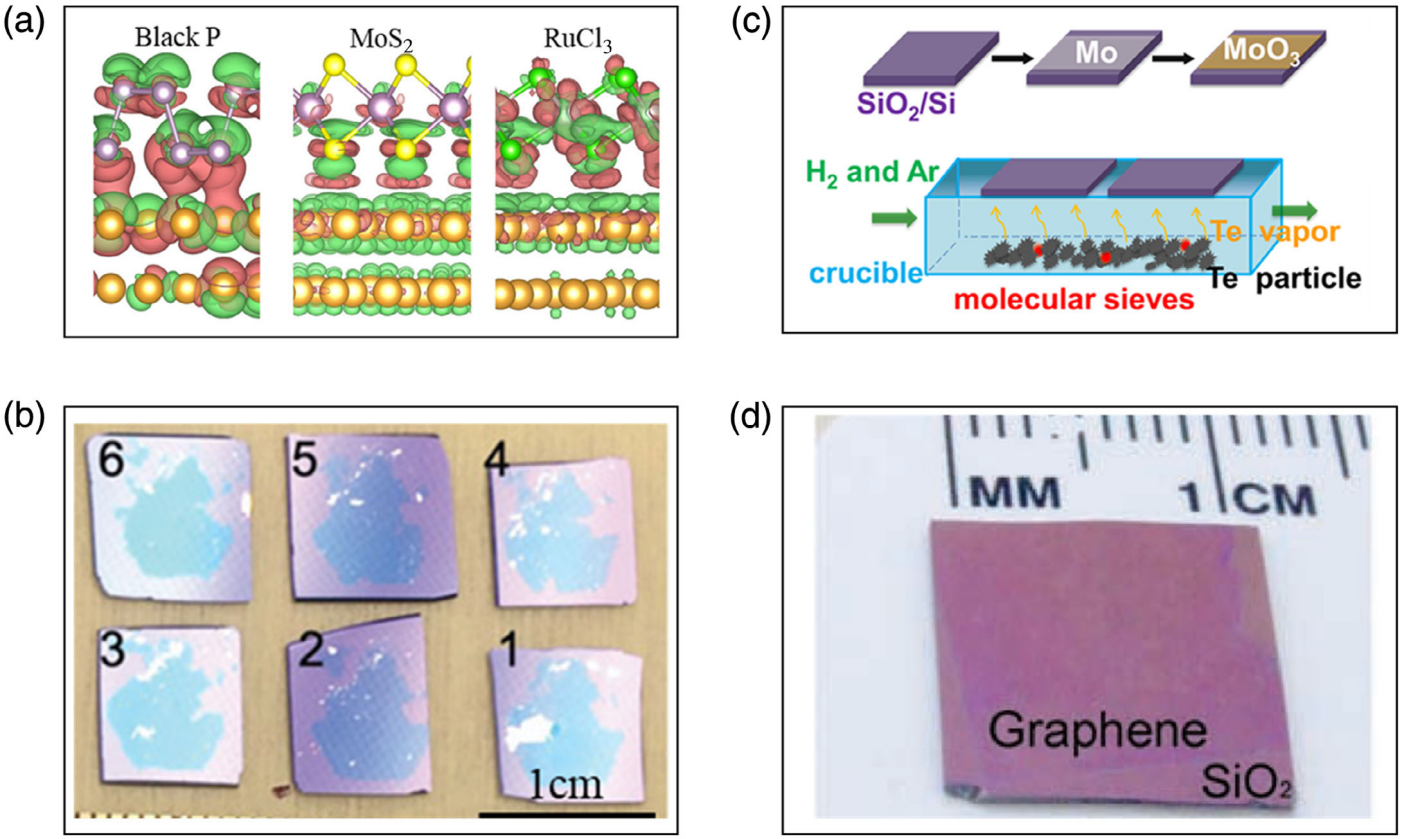
Exfoliated large‐area flakes prepared by mechanical exfoliation method and CVD method. a) Differential charge density (DCD) of four Au (111)/2D crystal interfaces with (nonmetallic) terminating atoms between groups 14 (IVA) and 17 (VIIA). Isosurface values of these DCD plots are 1 × 10^−2^ e Bohr^−3^ (BP), 1 × 10^−3^ e Bohr^−3^ (MoS_2_), and 1 × 10^−3^ e Bohr^−3^ (RuCl_3_), respectively. b) Optical images of six monolayer samples (on SiO_2_/Si substrate) sequentially exfoliated from a centimeter‐size WSe_2_ single crystal shown at the upper left corner. c) Experimental setup of the CVD system. d) Graphene films transferred onto a SiO_2_/Si substrate. a) Reproduced under the terms of the CC‐BY 4.0 license.^[^ ^63^ ^]^ Copyright 2020, The Authors, published by Springer Nature. b) Reproduced with permission.^[^ ^26^ ^]^ Copyright 2020, American Association for the Advancement of Science. c) Reproduced with permission.^[^ ^144^ ^]^ Copyright 2015, American Chemical Society. d) Reproduced with permission.^[^ ^44^ ^]^ Copyright 2009, American Association for the Advancement of Science.

**Figure 4:**
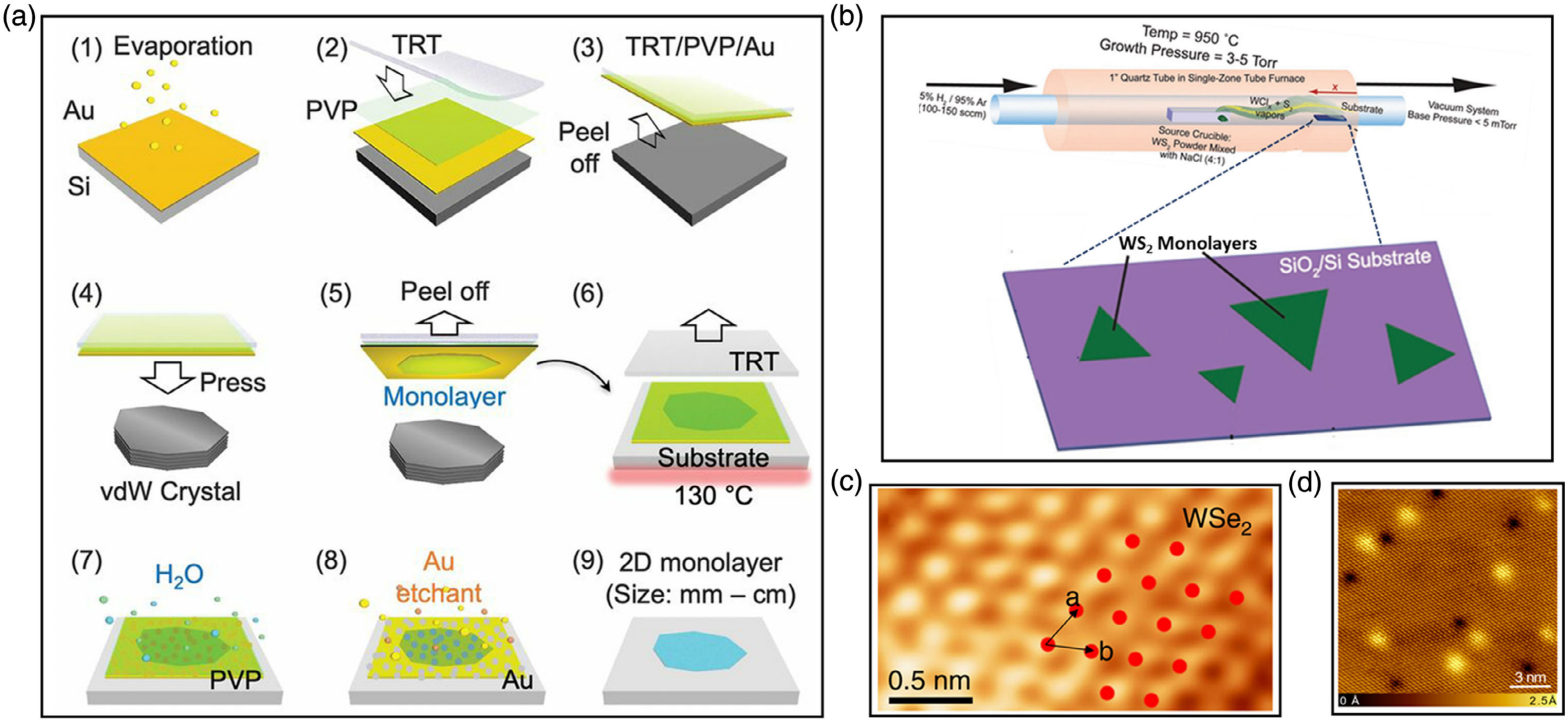
High‐quality flakes prepared by metal‐assisted exfoliation method and CVT method. a) Schematic illustration of the layer‐by‐layer exfoliation procedure of bulk vdW single crystals. Method: 1) Depositing gold on an ultraflat silicon wafer; 2) spin‐coating the surface with a layer of PVP; 3) using thermal release tape (TRT) to pick up the PVP and gold; 4) pressing the ultraflat gold onto the surface of a bulk vdW crystal; 5) peeling off a monolayer and transferring onto a substrate; 6) removing the TRT with heat; 7) dissolving PVP in water; 8) dissolving gold in an I_2_/I^−^ etchant solution; and 9) obtaining the single‐crystal monolayer with macroscopic dimensions. b) Schematic of open‐tube chemical vapor transport growth system. At the center of a single‐zone tube furnace, a crucible contains WS_2_ powder mixed with NaCl that is heated to 950 °C, forming tungsten chloride(s) and sulfur gases. The reverse process occurs downstream, resulting in monolayer WS_2_. c) STM images of monolayer WSe_2_. d) Defect atomic and electronic structure high‐resolution STM topographic images of MoSe_2_ (*V* = 1.25 V, *I* = 100 pA) showing a 25 nm area with two types of defects. a) Reproduced with permission.^[^ ^26^ ^]^ Copyright 2020, American Association for the Advancement of Science. b) Reproduced with permission.^[^ ^78^ ^]^ Copyright 2017, Wiley‐VCH. c) Reproduced under the terms of the CC‐BY 4.0 license.^[^ ^63^ ^]^ Copyright 2020, The Authors, published by Springer Nature. d) Reproduced with permission.^[^ ^74^ ^]^ Copyright 2019, American Chemical Society.

High‐quality 2D single crystals are the cornerstone for intrinsic properties of 2D materials, such as the quantum Hall effect, massless Dirac Fermions, superconductivity, and moiré excitons in TMD heterobilayers.^[^ 10, 66, 67, 73 ^]^ Point defects strongly cause carrier scattering and localization and may be responsible for large variation of electric and optical properties of TMD monolayers.^[^ ^74^ ^]^ Most of the aforementioned physical phenomena are observed on mechanical exfoliated 2D materials (**Figure** **3**) because of the highest quality monolayers prepared by mechanical exfoliation thus far. However, some physical phenomena are either suppressed or unobserved in samples obtained by other methods, such as liquid phase exfoliation and CVD.^[^ 75, 76 ^]^ As for liquid phase exfoliation, intercalation agent can be used to weaken the interlayer adhesion and increase the layer spacing. Then, the precipitate is removed to obtain homogeneous dispersed liquid after sonication and centrifugation. Therefore, there is often contamination of the isolated 2D surfaces, which causes poor quality.^[^ ^52^ ^]^ Metal‐assisted mechanical exfoliation is a common method to prepare high‐quality 2D crystals.^[^ 3, 16 ^]^ As shown in **Figure** **4**a, Liu et al. used an ultraflat gold film on a polymer substrate to contact 2D bulk crystals. After removing the polymer substrate and Au films, the quality of obtained monolayer crystals is comparable with or slightly better than that of single‐crystal monolayers using the traditional Scotch tape method.^[^ ^26^ ^]^ Recently, Huang et al. developed a contamination‐free, one‐step, and universal preparation strategy for high‐quality monolayer materials with Au films (Figure 4c).^[^ ^63^ ^]^ A freshly cleaved layered bulk crystal on tape is brought in contact with the Au layer deposited onto a substrate covered with a thin Ti or Cr adhesion layer to avoid chemical solvents. And the Au film can eliminate charging effects associated with insulating substrates. The sample can be used directly to fabricate devices to explore its physical properties. The chemical vapor transport (CVT) is a mature technology of nonvolatile solid crystallization by utilizing a transport medium (usually gas) to transport raw materials from a hot region to a cooler zone for crystallization (Figure 4b). The technology has been widely used in the crystal preparation of high‐quality 2D materials for decades.^[^ 77, 78, 79, 80 ^]^ Edelberg et al. enabled the highest quality MoSe_2_ and WSe_2_ crystals with defect densities lower than 10^11^ cm^−2^ based CVT method (Figure 4d).^[^ 60, 74 ^]^

### 2.2 Out‐of‐Plane Stacking Order

Apart from the intrinsic properties of the atomic‐thick Lego‐like basic building blocks of 2D materials, out‐of‐plane stacking order provides a second DOF of vdW materials to steer the optoelectronic properties of the artificial atomic lattices. By engineering the artificial interfaces and electronic band structures, the basic on‐chip components and/or devices such as atomic PN junctions and transistors can be flexibly constructed, beneficial for the next‐generation electronic integration beyond conventional semiconductor technologies.^[^ 74, 81, 82 ^]^ Also, the precise control of out‐of‐plane stacking order can provide alternative opportunity to investigate macroscopic quantum effects such as superconductivity, superfluidity, and Boson–Einstein condensation in much more simplified systems.^[^ 83, 84 ^]^ Note that basically well‐defined band alignment requires precisely out‐of‐plane stacking order of multiple layers and large lateral area of highly uniform structure, which requires high‐quality fabrication procedures. Therefore, two of the most important FOMs of potential electronic or optoelectronic devices are attainable number of heterostructures that can be vertically stacked and lateral uniformity of the heterostructures. Wrinkles, bubbles, and contaminants among interlayers should be minimized to improve the performance of devices.^[^ 85, 86, 87 ^]^ Thus far, many fabrication techniques have been developed to stack the desired structures. Later, we summarize the most promising methods toward high‐quality multilayer stacked heterostructures.

#### 2.2.1 Number of Vertical‐Stacking Layers

**Figure 5:**
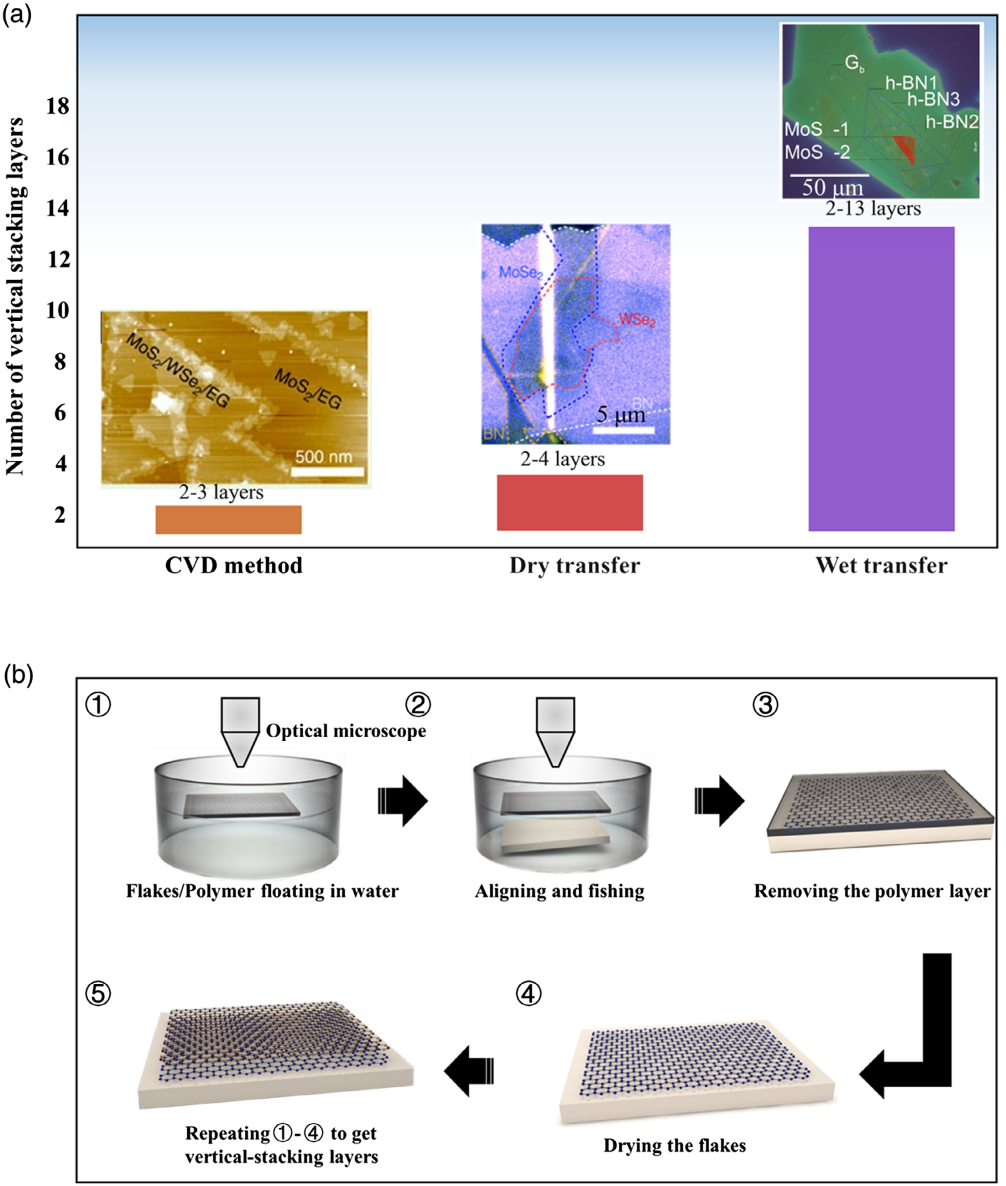
Multilayer heterostructures assembled by different methods. a) Several methods of assembling heterostructures in terms of the number of layers. b) Schematic of wet transfer. ➀ Flakes with polymer float in liquid. ➁ Align flakes and substrate and fish. ➂ Remove polymer. ➃ Dry the flakes. ➄ Repeating the above process to get vertical‐stacking layers. a) Left image: Reproduced under the terms of the CC‐BY 4.0 license.^[^ ^89^ ^]^ Copyright 2015, The Authors, published by Springer Nature. a) Middle image: Reproduced with permission.^[^ ^145^ ^]^ Copyright 2020, Springer Nature. a) Right image: Reproduced with permission.^[^ ^95^ ^]^ Copyright 2015, Springer Nature.

After the preparation of large‐area and thin 2D materials, precise positioning and vertical stacking of multiple atomic layers are critical in constructing homostructures or heterostructures, quantum wells, superlattices, and artificial atomic crystals, which depends on the number of stacking layers. Several methods of assembled vertical heterostructures manually are shown in **Figure** **5**a. Due to the abundance of 2D materials and relatively weak vdW‐like forces for stacking them together, there are no theoretical limitations on the attainable number of 2D vertical heterostructures. However, some realistic issues make it challenging to obtain multilayer heterostructures. The number of vertical stacking layers can be up to three layers so far by CVD, mainly limited by lattice mismatch among different 2D materials as well as substrates, or by the complexity of rational control of growth conditions.^[^ 56, 88 ^]^ In the past years, a variety of heterostructures have been prepared by CVD growth, such as graphene/TMD, graphene/TMD/TMD vertical heterostructures.^[^ 56, 89, 90, 91, 92 ^]^ 2D heterostructures are commonly a few layers fabricated by dry transfer methods, originating from that 2D sample on the substrate was not observed clearly with increasing the number of 2D sheets.^[^ 22, 93, 94, 95, 96 ^]^ A more convenient way to prepare multilayer heterostructures arbitrarily is stacking individual 2D building blocks by wet transfer method. Figure 5b shows a schematic of wet transfer. First, the transparent polymers with prepared 2D crystals are used as supporting layer that can float on the liquid. As the supporting layer, polymer protects 2D materials from shrinking during the transfer process, and ensures the successful transfer of target 2D materials, and then, with a microscope, aligns the 2D crystals and the substrate by pumping down the water. To align the flake onto the target substrate, a needle coupled to a micromanipulator can be used to position the floating polymer at will gently. Finally, the polymer layer is removed by dissolving it into a solvent. A complex vertical structure can be obtained by repeating the aforementioned steps continuously. The self‐floating polymer‐loaded 2D crystals are prepared with different methods, such as the substrate etching and wedging methods. The sacrificial layer etching method is achieved by etching the spacing material film (such as silicon dioxide on silicon wafer) between the sample and the substrate. However, it does not work for special substrates such as Ir and Pt due to lack of cost‐effective or effective wet‐etching etchant.^[^ ^39^ ^]^ Gurarslan et al. developed wedging method due to different surface energies to separate the polymer from the substrate.^[^ ^97^ ^]^ The self‐floating sample makes the method rather challenging in precise positioning the building blocks during the vertical stacking process. Therefore, it is time‐consuming and certain degree of operational proficiency is required.^[^ ^37^ ^]^ Recently, an automated searching system has attracted a lot of attention from researchers.^[^ 36, 98, 99, 100, 101, 102, 103 ^]^ The automated searching system provided very favorable opportunities for exploring the full potential of 2D vertical heterostructures, resulting in being no longer limited by manual tasks.

#### 2.2.2 Lateral Uniformity of Stacked Structure

**Figure 6:**
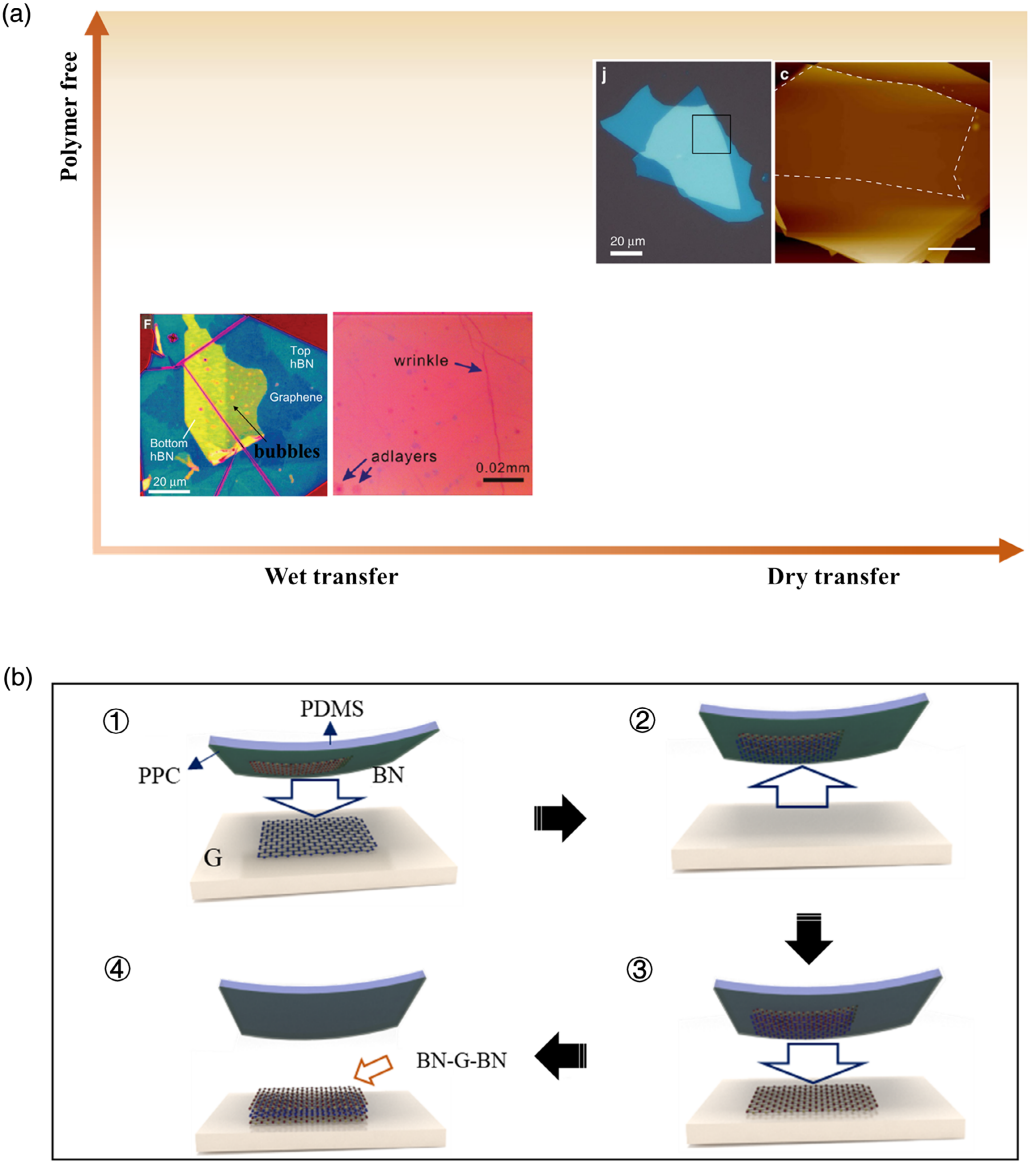
Transfer methods with different cleanliness. A) Cleanliness of dry transfer and wet transfer. b) Schematic of the vdW technique for polymer‐free assembly of layered materials. a) Bottom left image: Reproduced with permission.^[^ ^146^ ^]^ Copyright 2015, American Association for the Advancement of Science. a) Bottom right image: Reproduced with permission.^[^ ^147^ ^]^ Copyright 2011, American Chemical Society. a) Top left image: Reproduced under the terms of the CC‐BY 4.0 license.^[^ ^24^ ^]^ Copyright 2016, The Authors, published by Springer Nature. a) Top right image: Reproduced under the terms of the CC‐BY 4.0 license.^[^ ^25^ ^]^ Copyright 2018, The Authors, published by Springer Nature.

Once a certain number of Lego‐like basic building blocks of 2D materials (2D material monolayers or flakes) are assembled vertically, the electronic or optoelectronic properties of the overall structure can still be more or less deviate from the ideal 2D flakes based on Lego because there may be other undesired uncertainty that may introduce lateral uniformity such as cracks, wrinkles, bubbles, and surface cleanness. Lateral spatial uniformity may change electric conductance, environmental refractive index, and thermal insulation, which deteriorate almost all kinds of devices. Qualitative comparison in terms of cleanliness for constructing heterostructures is shown in **Figure** **6**a. Wet transfer is a process of continuous picking up and dropping 2D sheets. In the post process, chemical reagents are often used for polymer removal, which leads to inhomogeneities caused by polymer residues between layers. And adsorbates tend to form relatively bubbles of the trapped material, which are clearly seen in an optical microscope.^[^ 104, 105 ^]^ Annealing treatment is the universal way to remove bubbles and polymer residues. Kinetic energy generated by high temperature would drive small bubbles gathering with each other and form a big bubble, and then part of bubbles would escape from the surface edges.^[^ ^106^ ^]^ However, polymer residues between the interfaces may not be completely removed by postannealing. Pizzocchero et al. developed vdW pickup transfer process in building heterostructures, which is a continuous picking up 2D crystal process. The interfacial adhesion and cleanliness vary with temperature, completely avoiding any contact with polymer and chemical solvents in the stacking procedure. On heating up to ≈100 °C, bubbles (and the contaminants trapped inside) become physically mobile, enabling them to be pushed to the heterostructure edges and eliminated.^[^ 107, 108 ^]^ The detailed steps of this method are shown in Figure 6b. First, a polymer film such as PPC is spinning coating onto PDMS surface, and these two layers are stuck on a glass side which is attached to a micromanipulator for precise positioning. The interface adhesion is strong at 40 °C so as to pick up 2D materials continuously. When the pickup process is over, the heterostructure assembled can be dropped down to the target substrate as the temperature is raised to 110 °C.^[^ ^24^ ^]^ Purdie et al. reported blister‐free regions of graphene encapsulated in h‐BN with an area of ≈5000 μm^2^ by vdW pickup transfer method.^[^ ^25^ ^]^

### 2.3 in‐Plane Twist‐Angle Alignment

#### 2.3.1 Controlling Twist Angle in the Macroscopic Level

**Figure 7:**
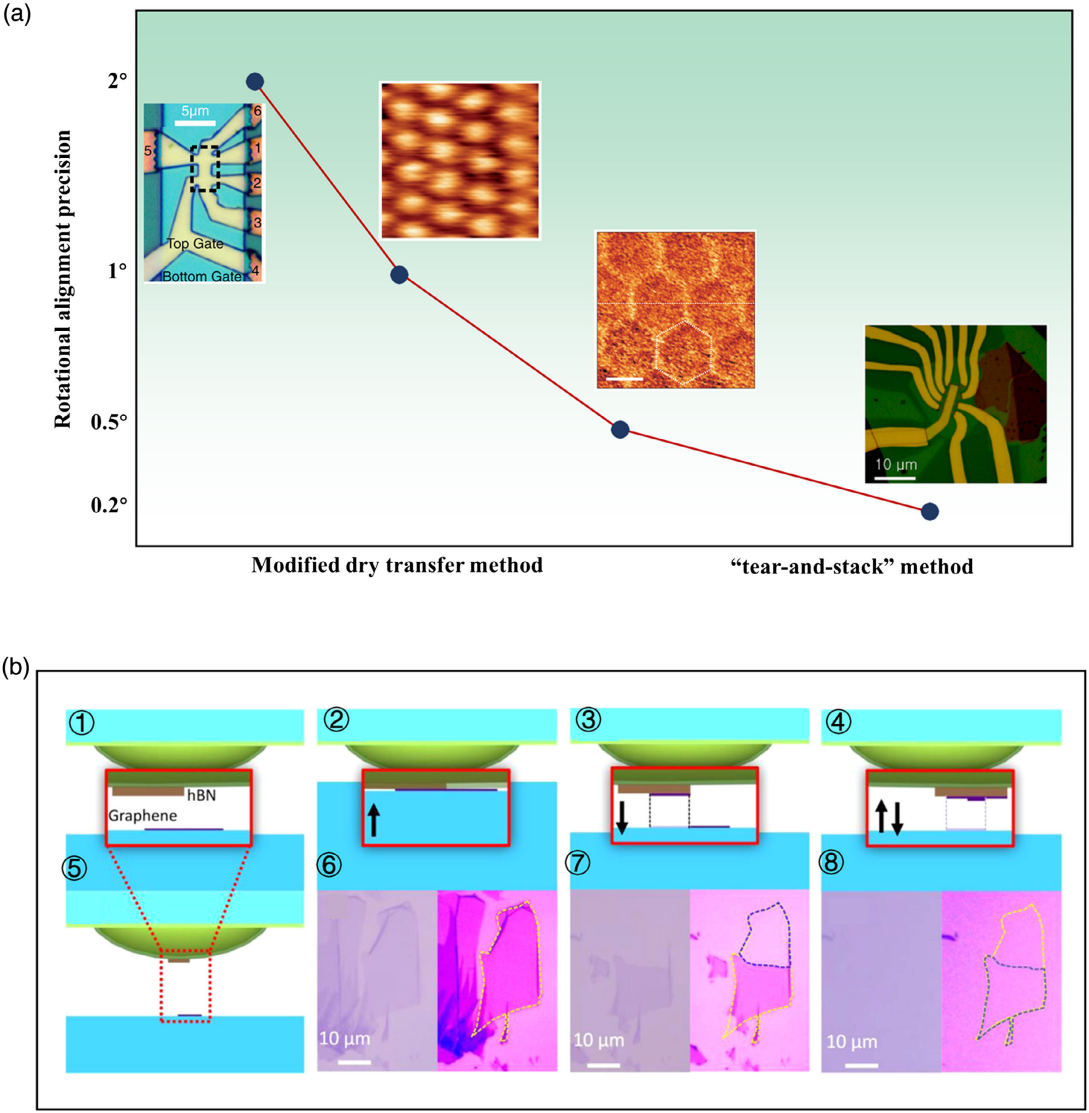
Schematics of the in‐plane twist‐angle alignment process stacking. a) Precise control of twist angle. b) Schematic of layer pickup. The red box represents a zoom‐in view of the hemispherical handle substrate (➀,➄). (➁‐➃) and (➅‐➇) Corresponding optical micrographs of successive stacking steps. Panels ➁ and ➅ illustrate a partial contact of the handle with the bottom graphene. Panels ➂ and ➆ show the handle substrate release with one graphene section detached. Panels ➃ and ➇ illustrate the second contact with the handle translated laterally to create an overlap region of the two graphene layers. The two images in panels ➅‐➇ represent the same optical micrographs, with the contrast enhanced and the graphene contour marked in the right‐hand image. Because the two graphene layers are obtained from a single grain, their principal crystal axes remain aligned as long as the handle is not rotated with respect to the bottom substrate. a) Left image: Reproduced with permission.^[^ ^119^ ^]^ Copyright 2016, American Physical Society. a) Second image from left: Reproduced with permission.^[^ ^109^ ^]^ Copyright 2013, Springer Nature. a) Second image from right: Reproduced with permission.^[^ ^118^ ^]^ Copyright 2014, Springer Nature. a) Right image: Reproduced with permission.^[^ ^120^ ^]^ Copyright 2017, National Academy of Sciences. b) Reproduced with permission.^[^ ^28^ ^]^ Copyright 2016, American Chemical Society.

As a new DOF, in‐plane twist‐angle alignment is the twist angle between the atomic/crystalline lattices of the individual layers in a vdW stacking. Manipulating the “twist angle” between the two layers enables fine control of the electronic band structure, resulting in magic‐angle fat‐band superconductivity, the formation of moiré excitons, the Hofstadter spectrum, and interlayer magnetism.^[^ 109, 110, 111, 112 ^]^ It is crucial to control the accuracy of twist angle. After obtaining 2D materials, nonlinear optical techniques can be applied to identify the crystallographic orientation in 2D crystals successfully, such as second harmonic generation (SHG) and polarized Raman.^[^ 113, 114, 115, 116 ^]^ As for SHG technique, SHG signals cannot be directly identified such as graphene and isotropic 2D crystals including most TMDs. Recently, Cui et al. developed a chemical method to realize the visualization of crystallographic orientation with an optical microscope.^[^ ^117^ ^]^ The schematics of in‐plane twist‐angle alignment precise is shown in **Figure** **7**a. The control of macroscopic twist angle has been equal or smaller than 0.2° up to now.^[^ 58, 118, 119, 120, 121, 122, 123 ^]^ The “tear‐and‐stack” technique is favorable to control the stacking angle of layered materials.^[^ 28, 108, 124, 125, 126, 127 ^]^ The fabrication process of “tear‐and‐stack” technique is shown in Figure 7b. Sample stage of transfer setups can be rotated to make the top and bottom 2D flakes aligned at a certain angle. Using this technique, Cao et al. and Kim et al. fabricated twisted bilayer graphene (TBLG) devices to observe insulating states induced by strong interlayer interactions via superlattice modulation.^[^ 119, 120 ^]^ Chen et al. utilized vdW force between scanning tunnelling microscopy (STM) tip and graphene to realize arbitrary twist angle from 0° to 60°, enabling various folding directions with an accuracy of 0.1°.^[^ ^110^ ^]^ The fabrication process of twisted graphene strongly relies on manual operation, limited by the artificial uncertainty.^[^ 127, 128 ^]^ An efficient automated twist‐angle stacking system is indispensable with higher accuracy.

#### 2.3.2 Precise Control of Twist Angle in the Microscopic Level

**Figure 8:**
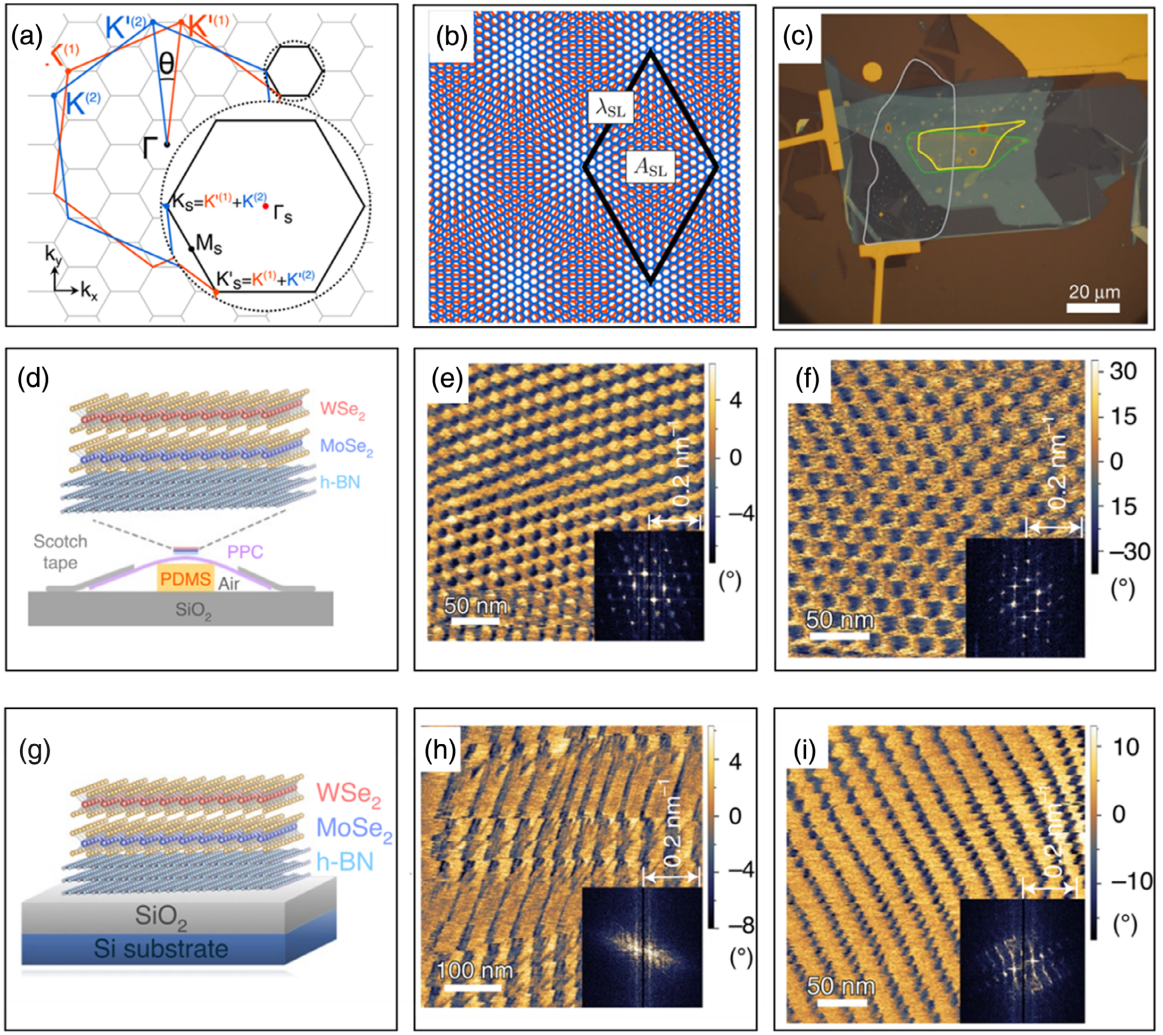
Precise control of in‐plane twist‐angle alignment at the microscopic level. a) The orange and blue hexagons denote the original Brillouin zones of graphene layer 1 and 2, respectively. In *k* space, the band structure is folded into the mini Brillouin zone (MBZ) which is defined by the mismatch between the hexagonal Brillouin zones of the two honeycomb lattices. b) Schematic of TBLG and its superlattice unit cell. *λ*_SL_={ *a*/[2 sin(*θ*/2) ] (*a* is the lattice constant of graphene) is the moiré period and ASL=$(\sqrt{3}/2)\lambda_{SL}^{2}$ is the unit cell area. c) Optical microscopy image of a heterostructure with near‐zero in‐plane twist‐angle alignment. The graphite top gate, WS_2_, and WSe_2_ flakes are outlined in gray, yellow, and green, respectively. d,g) Schematics of the h‐BN/WSe_2_/MoSe_2_ stack, without the top h‐BN capping layer and with Δ*θ* = 59.4 ± 0.8°, on air‐cushioned polypropylene carbonate (PPC) tape (d) and the Si/SiO_2_ surface (g). e,f) Piezoresponse force microscopy (PFM) images at three locations before transfer of the stack from the PPC tape to the Si/SiO_2_ substrate. h,i) PFM images at three locations after transfer of the stack from the PPC tape to the Si/SiO_2_ substrate. Insets: Fast Fourier transforms (FFTs) of the PFM images. a,b) Reproduced with permission.^[^ ^119^ ^]^ Copyright 2016, American Physical Society. c) Reproduced with permission.^[^ ^148^ ^]^ Copyright 2020, Springer Nature. d‐i) Reproduced with permission.^[^ ^145^ ^]^ Copyright 2020, Springer Nature.

The control of interlayer twist angle in 2D vdW heterostructures enables one to manipulate moiré superlattice for different moiré patterns with superlattice period ranging from a few nanometers up to hundreds of nanometers.^[^ 129, 130, 131, 132, 133 ^]^ In Section 2.3.1, methods of controlling twist angle in the macroscopic level are summarized. However, the transport or scanning probe measurements were conducted for no real control over the twist angle in the fabrication so far. It has been found samples on the hard substrate with residual strain, leading to the distortion and distortion of the moiré pattern in **Figure** **8**g–i in the past researches.^[^ ^125^ ^]^ Recently, Bai et al. used a soft material as substrate, such as PDMS, to effectively release the strain inside the sample for producing more uniform moiré pattern (Figure 8d‐f).^[^ ^73^ ^]^ Recently, Søren Ulstrup et al. linked mesoscale structural variations with electronic states with nanoscale spatial resolution (nanoARPES).^[^ ^134^ ^]^ The study showed that a range of twist angles was from 9.8° to 12.7° on a length scale of 2 μm of TBLG, which further illustrated the distortion and inhomogeneity in the TBLG at the microscopic scale.

## 3 Challenges and Perspectives

In recent years, researchers have developed various techniques for manipulating the DOF of 2D materials, paving the pathway toward rich physics and high‐performance 2D‐based devices. Although great advancements have been made as overviewed earlier, we stress that many factors need to be fully considered for constructing an ideal 2D heterostructure due to the correlation between different DOFs, such as the types of devices, the crystal quality and sources of 2D materials, and so on. There are always tradeoff issues for different fabrication processes in terms of precise control of different DOFs. For fundamental research, the ideal samples can be obtained by combining high‐quality crystal and clean transfer method, such as mechanical exfoliation and modified dry transfer due to the small constraint of sample size. For industrial applications, CVD and roll‐to‐roll transfer method are used to producing large‐scale 2D materials and heterostructures, but resulting in compromised quality. Building up a bridge to fill the gap between the fields is of great importance in the future. An ideal strategy to assemble a uniform vertical stacking heterostructure could be a polymer‐free transfer method and/or a completely clean polymer removing method. When the transfer process changes from face‐to‐face contact to point‐to‐point contact, it can minimally reduce the introduction of polymer and even have no polymer residues at all by using mechanical contact scribing.^[^ 135, 136, 137 ^]^ Based on the above, mechanical contact scribing will be developed into a common polymer‐free transfer method if it were easier to operate. As for the control of out‐of‐plane stacking order and in‐plane twist‐angle alignment, postannealing treatment is often used to eliminate or relieve internal strain. A friendly strain‐free stacking method will be highly desired especially for researches on intrinsic optoelectronic behaviors in the future. However, it does not mean that strain engineering on the 2D heterostructures makes no sense. Indeed, it is also of potential applications to make use of the strain caused by the fabrication process of 2D materials and/or 2D heterostructures, such as wrinkles, bubbles, and so on.^[^ 61, 138, 139 ^]^ Under external strain, electronic band structure of materials, including the magnitude and type of bandgaps, would be changed by the stretched or compressed lattice structure of 2D materials, leading to modulate the electrical and optical properties.^[^ ^139^ ^]^ Especially for 2D materials with strain sensitivity, such as MoS_2_, it is ideal to achieve modulations of photoelectric performance by strain engineering.^[^ 140, 141, 142 ^]^

## Conflict of Interest

The authors declare no conflict of interest.

## Author Contributions

H‐Z.Z. and W‐J.W. contributed equally to this work. H.‐Z.Z.: Writing—original draft, writing—review and editing. W.‐J.W.: Writing—review and editing. L.Z.: Conceptualization, supervision, writing—review and editing. Z.W.: Writing—review and editing. J.Z.: Supervision, conceptualization, writing—review and editing.
